# A systematic review of factors affecting intended and actual adherence with antiviral medication as treatment or prophylaxis in seasonal and pandemic flu

**DOI:** 10.1111/irv.12406

**Published:** 2016-08-08

**Authors:** Louise E. Smith, Donatella D'Antoni, Vageesh Jain, Julia M. Pearce, John Weinman, G. James Rubin

**Affiliations:** ^1^ Department of Psychological MedicineKing's College LondonLondonUK; ^2^ Institute of Pharmaceutical ScienceKing's College LondonLondonUK; ^3^ Department of War StudiesKing's College LondonLondonUK

**Keywords:** adherence, antivirals, influenza, pandemic

## Abstract

The aim of this review was to identify factors predicting actual or intended adherence to antivirals as treatment or prophylaxis for influenza. Literature from inception to March 2015 was systematically reviewed to find studies reporting predictors of adherence to antivirals and self‐reported reasons for non‐adherence to antivirals. Twenty‐six studies were included in the review; twenty identified through the literature search and six through other means. Of these studies, 18 assessed predictors of actual adherence to antivirals, whereas eight assessed predictors of intended adherence. The most commonly found predictor of, and self‐reported reason for, non‐adherence was the occurrence of side effects. Other predictors include perceptions surrounding self‐efficacy, response efficacy and perceived personal consequences as well as social influences of others' experiences of taking antivirals. Predictors identified in this review can be used to help inform communications to increase adherence to antivirals in both seasonal and pandemic influenza.

## 1 Introduction

In April 2009, a new strain of influenza virus was detected in humans (influenza A(H1N1)pdm09). The illness, commonly referred to as “swine flu,” was first identified in Mexico and spread rapidly around the globe. The World Health Organization (WHO) declared pandemic phase 5 on 29 April 2009 and raised this alert to pandemic phase 6, the highest of the WHO pandemic ranks, on 11 June 2009.

Oseltamivir phosphate (Tamiflu^®^) is an antiviral medication that is used as prophylaxis and treatment for influenza, and was prescribed widely during the 2009/2010 swine flu pandemic. As a treatment, it does not cure swine flu, but shortens the duration of symptoms.^1^, ^2^ Within the United Kingdom, total expenditure on oseltamivir was estimated to be £160.4 million in 2009–2010, with 2.4 million units of oseltamivir “consumed” between 2009/2010 and 2012/2013, the majority of these during the pandemic.^3^

In an effort to gauge oseltamivir adherence in one area of the UK during the pandemic, Singer et al.^4^ measured levels of the active metabolite of oseltamivir in wastewater. When comparing these levels with the number of prescriptions collected, adherence was estimated to be in the range of 45%–60%. The repercussions of non‐adherence to antiviral medication are widespread, including the monetary cost of unused antivirals,^4^ a longer duration of absenteeism from work and the potential implication of more drastic measures such as school closures, household quarantine and restrictions on travel. In the UK, the cost of school closure alone is estimated at £0.2–£1.2 billion.^5^ Other health implications of non‐adherence to antivirals include possible increase in future antiviral resistance and the possibility of misuse leaving a shortage of supply.^4^

There are many reasons why people may choose not to adhere to their antiviral medication as prescribed. Research into medication adherence has suggested that reasons for non‐adherence include social and economic factors (such as social support, family/caregiver factors and socio‐economic status), therapy‐related factors (such as the presence of adverse effects, duration of treatment and drug effectiveness), patient‐related factors (such as age, gender and education) and condition‐related factors (such as the presence of symptoms and disease severity), amongst others.^6^ To the best of our knowledge, there is no systematic review describing factors affecting adherence to antiviral medication for influenza specifically.

Given that adherence is a complex behavioural process,^7^ the successful implementation of interventions that promote adherence requires a thorough understanding of the factors associated with that behaviour. The Capability, Opportunity and Motivation (COM‐B) framework was developed from existing theories of behaviour change.^8^ In essence, the framework hypothesises that the interaction between capability (C), opportunity (O) and motivation (M) causes the performance of behaviour (B) and can provide a framework for understanding why a desired or recommended behaviour is not performed. *Capability* is defined as the individual's psychological and physical capacity to engage in the activity and includes having the necessary knowledge and skills; *Opportunity* is defined as all the factors that lie outside the individual that make the behaviour possible or prompt it; and *M*o*tivation* is defined as the mental processes that energise and direct behaviour, not just goals and conscious decision‐making but also habitual processes and emotional responses. The COM‐B approach has recently been applied to medication adherence.^9^ It is able to account for a wide range of factors affecting adherence and to inform behaviour change interventions that can be used to guide healthcare practitioners involved in the care of non‐adherent patients.

To inform policy regarding the distribution of and communication about antivirals as treatment or prophylaxis in seasonal and future pandemic influenza, we conducted a systematic review to investigate factors associated with adherence and non‐adherence to antiviral medication for influenza. The outcome measures of this review included predictors of adherence and non‐adherence, and self‐reported reasons for non‐adherent behaviours.

## 2 Method

The review was conducted in accordance with the PRISMA guidelines,^10^ using systematic methods to identify and select studies, and assess their risk of bias. No formal protocol exists for this review.

### 2.1 Search strategy

We searched EMBASE, MEDLINE, PsycINFO and the Web of Science Core Collection from inception to 26 March 2015, using combinations of terms relating to antivirals (e.g. antiviral, Tamiflu, oseltamivir, Relenza, zanamivir), influenza (e.g. pandemic, influenza, H1N1, H5N1) and adherence (e.g. adherence, uptake, compliance). There was no limit on publication date imposed. Where possible, databases were also searched using MeSH headings. We also undertook reference tracking to identify further papers for inclusion. Other articles were located through previous non‐systematic searches carried out by members of the research team.

### 2.2 Inclusion criteria

Eligibility criteria for inclusion in the systematic review were as follows:

Participants: Studies were included if they asked people whether they took antivirals as either treatment or prophylaxis for influenza or whether they intended to take antivirals as treatment or prophylaxis for influenza. Participants could be drawn from the general public, patient groups or specific occupational groups.

Predictors/Exposures: We only included studies if they assessed demographic or psychosocial predictors of adherence to antivirals in the context of influenza or if they assessed self‐reported reasons for adherence or non‐adherence to a course of antivirals.

Outcomes: Studies were included if they reported actual or intended adherence to antivirals, which were intended as either treatment or prophylaxis for pandemic, avian or seasonal influenza.

Study reporting: All study designs, aside from those published only as conference papers, editorials or abstracts, were included. For pragmatic reasons, we only accepted papers published in English.

### 2.3 Data extraction

For every included paper, we tabulated details relating to the author, date of publication, influenza virus, country, sample size, methodology, adherence with antivirals, length of course of antivirals, and reasons for, and predictors of, non‐adherence.

### 2.4 Data synthesis

Where possible, we grouped study results together depending on whether they related to actual vs intended adherence, antivirals prescribed as a treatment vs antivirals prescribed as a prophylaxis and pandemic (influenza A(H1N1)pdm09) vs non‐pandemic situations. In practice, it was not always possible to make these subdivisions due to a limited amount of data being available.

A meta‐analysis of the data was not planned, because we expected that the literature would be too heterogeneous. Instead, we carried out a narrative synthesis of the data. No general consensus exists on the best methodological approach to the narrative synthesis of data.^11^ In this review, we created a comprehensive list of predictors of actual and intended adherence to antivirals that have been studied to date. Effect sizes were not synthesised.

### 2.5 Risk of bias

Risk of bias was determined according to an adaptation of the Scottish Intercollegiate Guidelines Network (SIGN) critical appraisal methodology checklist for cohort studies^12^ and supplemented by relevant items from the Cochrane Collaboration's Risk of Bias tool.^13^ Table 1 shows the criteria used in the assessment.

**Table 1:** Criteria for risk of bias assessment

Code	Bias	Item(s)
A	Selection bias	The study indicates how many of the people asked to take part did so
		A clear definition of source of population and clear eligibility criteria for selection of subjects are used, to ensure the sample is representative
B	Detection bias	The outcomes are clearly defined
		The method of outcome assessment is valid and reliable
C	Reporting bias	Confidence intervals have been provided
		Appropriate statistical analyses have been carried out
		The main potential confounders are identified and taken into account in the design and analysis
D	Other bias	A power calculation is reported. If not, sample size is small, medium or large (Small, n=30–59; medium, n=60–150; large, n=150+)
		The study addresses an appropriate and clearly focused question

### 2.6 Procedure

LS and VJ developed and conducted our literature search. Studies were screened by LS, and data extraction, assessment of risk of bias and data synthesis were carried out independently by LS and DD with advice from JP, GJR and JW. Any discrepancies were resolved through discussion.

## 3 Results

**Figure 1:**
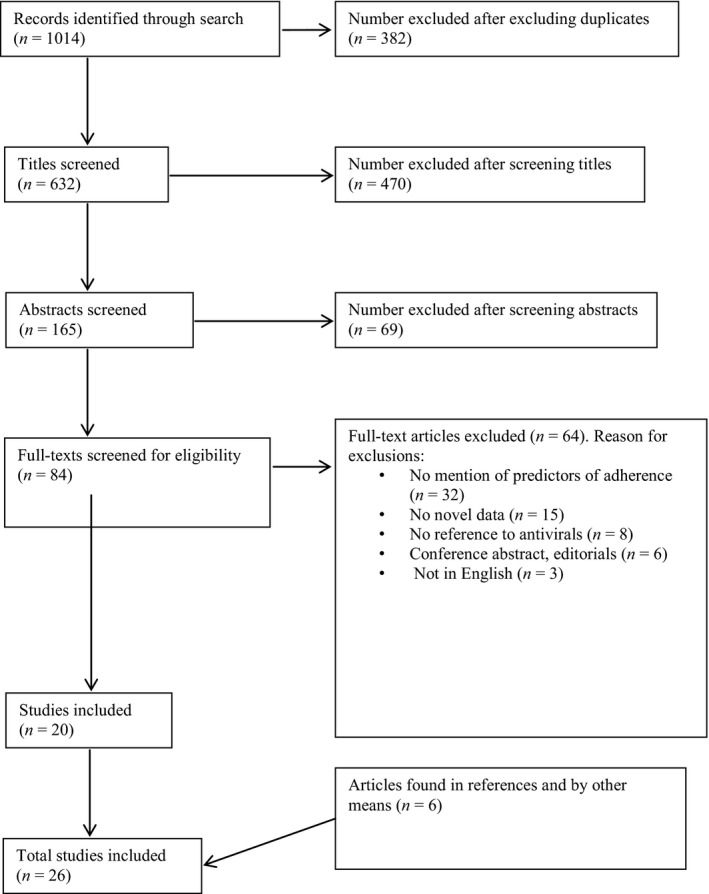
Literature search

Figure 1 illustrates the results of our literature search. We identified 1014 citations through our database search, of which twenty fulfilled the inclusion criteria. Six additional articles were identified through other search engines such as ScienceDirect and in review articles on the topic area. For one of our included studies,^14^ additional information was obtained from the author (Phern‐Chern, personal communication).

### 3.1 Study characteristics

Eighteen articles assessed actual adherence to antiviral medication, while eight assessed intended adherence. The studies spanned thirteen countries (Australia, Brunei, Canada, Cyprus, Hong Kong, Israel, Japan, Madagascar, Netherlands, Norway, UK, USA and Singapore) and included samples of healthcare workers, military personnel, pregnant or recently pregnant women, poultry farmers, end‐stage renal patients, school children and staff as well as the general public. All but three studies investigated pandemic influenza (actual influenza A(H1N1)pdm09 or hypothetical scenarios), with other studies investigating influenza H5N1,^15^ H7N3^16^ and seasonal influenza.^17^ The majority of studies were cross‐sectional, with one RCT,^18^ and two qualitative studies.^19^, ^20^ All but one study^18^ investigated adherence with antivirals via self‐report. All studies investigated predictors of actual or intended adherence to oseltamivir; no studies included in the review investigated predictors of adherence to zanamivir (trade name “Relenza”) or other antivirals such as amantadine. Tables 2 and 3 provide information about the samples and methods of the included studies, which addressed actual and intended adherence, respectively. Tables 4 and 5 provide adherence rates in each study, together with self‐reported reasons for, and predictors of, actual and intended non‐adherence. Where reported by individual studies, numerical data for significant predictors of adherence are stated in Tables 4 and 5.

**Table 2:** Methods of included studies measuring actual adherence

Citation	Study design	Virus	Location (time of data collection)	Number of participants (mean age, y)	Population (% of sample male)	Treatment/prophylaxis (course length)
Belmaker et al.^15^	Cross‐sectional study	H5N1	Southern Israel (March 2006)	N=201 (34.9)	Poultry farmers (98.5%)	Prophylaxis (until 7 d after last exposure)
Chishti & Oakeshott^32^	Cross‐sectional study	Pandemic influenza A(H1N1)	Inner London (December 2009–March 2010)	N=33 (27)	Primary care patients prescribed oseltamivir (55%)	Not reported (5 d)
Choo et al.^38^	Cross‐sectional study	Pandemic influenza A(H1N1)	Brunei Darussalam (June 2009–January 2010)	N=331 (52.0)	End‐stage renal disease patients of all dialysis centres in Brunei (47.1%)	Prophylaxis (1 mo)
Coleman et al.^18^	Pilot, randomised control trial of vaccine vs antivirals	Pandemic influenza A(H1N1)	Canada (October 2007)	N=56 (42)	Healthcare workers—employees and students (32.1%)	Prophylaxis (20 wk)
Griffiths et al.^39^	Cross‐sectional study	Pandemic influenza A(H1N1)	Hong Kong (July 2009)	N=359 (21)	Students attending an international summer school (51.8%)	Prophylaxis (not reported)
Kimberlin et al.^33^	Cross‐sectional study	Pandemic influenza A(H1N1)	Alabama, USA (July 2009)	N=75; 48 camp counsellors, 27 camp staff (not reported)	All staff and counsellors of a boys' camp in Mentone, Alabama with influenza pandemic outbreak (not reported)	Prophylaxis (10 d)
Kitching et al.^23^	Cross‐sectional study	Pandemic influenza A(H1N1)	London, UK (May 2009)	N=103 (not reported)	School children in the same class as a confirmed pandemic influenza case (not reported)	Treatment (5 d); prophylaxis (10 d)
Koliou et al.^24^	Cross‐sectional study	Pandemic influenza A(H1N1)	Nicosia, Cyprus (July–August 2009)	N=45 (median age, 9 y)	Laboratory‐confirmed cases of A(H1N1) in children aged under 16 (not reported)	Treatment (not reported)
Lonsdale & Way^25^	Cross‐sectional study	Pandemic influenza A(H1N1)	Lancashire, UK (August–September 2009)	N=188 (not reported)	Patients who had received antivirals at one of the collection points within NHS North Lancashire (not reported)	Treatment (5 d)
McVernon et al.^34^	Cross‐sectional study	Pandemic influenza A(H1N1)	Melbourne, Australia (November 2009)	N=314 (not reported)	Families of children whose schools had been closed (not reported)	Treatment (not reported); prophylaxis (not reported)
Rajatonirina et al.^28^	Cross‐sectional study	Pandemic influenza A(H1N1)	Antananarivo, Madagascar (November 2009)	N=132 (16.9)	Pupils attending boarding school (46.2%)	Treatment (5 d); prophylaxis (10 d)
Simonsen et al.^37^	Cross‐sectional study	Pandemic influenza A (H1N1)	Hordaland County, Norway (October–December 2009. NB Patients were sent questionnaire 2–4 wk after first encounter with GP's office)	N=357 (0–17 y, 30%; 18–40 y, 36%; >40 y, 34%)	People given influenza diagnosis in GP's records at selected practices (41%)	Not reported (not reported)
Skowronski et al.^16^	Cross‐sectional study	Avian influenza (H7N3)	British Colombia, Canada (May–July 2004)	N=167 (median age 34)	Anyone—cullers, farmers and their families—involved in efforts to control poultry outbreak (53%)	Prophylaxis (6+ wk)
Strong et al.^29^	Cross‐sectional study	Pandemic influenza A(H1N1)	Sheffield, UK (June 2009)	N=355, staff, n=58; students, n=297 (Students = 9.5. Staff, 64% (37/58) were aged between 40–59)	Staff and students in a school with an outbreak of pandemic influenza (Students, 55.2%; staff, 9.4). NB—only 341/355 indicated sex)	Treatment (5 d); prophylaxis (10 d)
Upjohn et al. 30	Cross‐sectional study	Pandemic influenza A(H1N1)	Melbourne, Sydney (“5 mo after pandemic”)	N=37 (not reported)	Health care workers offered oseltamivir as post‐exposure prophylaxis (41%)	Prophylaxis (10 d)
Ushijima et al.^17^	Cross‐sectional study	Seasonal influenza	Fukuoka Prefecture, Japan (January–February 2006)	N=299 (16.5)	Individuals prescribed oseltamivir, who collected prescription from one of seven pharmacies (not reported)	Treatment (2–5 d)
Van Velzen et al.^36^	Cross‐sectional study	Pandemic influenza A(H1N1)	Scotland, UK (May 2009)	N=108 (4.1)	Classmates of a confirmed pandemic influenza case (62%)	Prophylaxis (9–10 d); treatment (5 d)
Wallensten et al.^31^	Cross‐sectional study	Pandemic influenza A(H1N1)	South West England, UK (May 2009)	N=248 (not reported)	Same school year as a child with confirmed pandemic influenza (48.8%; one person not specified)	Prophylaxis (10 d)

**Table 3:** Methods of included studies measuring intended adherence

Citation	Study design	Virus	Location (time of data collection)	Number of participants (mean age in years)	Cohort (% of sample male)
Bults et al.^21^	Web based cross‐sectional surveys with one follow‐up survey	Pandemic influenza A(H1N1)	Netherlands (April–August 2009)	Survey 1 (May 2009), n=456; Survey 2 (June 2009), n=478; Follow‐up of survey 1 & 2 (August 2009), n=934 (Age at follow‐up: 18–29 y, 12.3%; age 30–49 y, 36.3%; 50+ y, 52%)	Dutch adult population—general public (52%)
Ibuka et al.^22^	Cross‐sectional study	Pandemic influenza A(H1N1)	USA (April–May 2009)	N=1290 (18–29 y, 13%; 30–39 y, 19%; 40–49 y, 21%; 50–64 y, 28%; 65+, 18%	US adult population—general public (49%)
Lynch et al.^20^	Qualitative, focus groups	Pandemic influenza A(H1N1) and seasonal influenza	USA (September 2009)	N=144 (18–24 y, 26%; 25–34 y, 62%; 35–44 y, 12%)	Pregnant or recently pregnant—within 6 mo post‐partum—women (0%)
Masuet‐Aumatell et al.^26^	Cross‐sectional study	Pandemic influenza A(H1N1)	London, UK (September 2009)	N=100 (32)	Travellers attending a UK travel clinic (43.8%)
Yap et al.^14^; Phern‐Chern, personal communication	Cross‐sectional study	Pandemic influenza A(H1N1)	Singapore (August–October 2009)	N=1063 (21.4)	Singapore military: 4.4% laboratory‐confirmed cases; 23.0% contacts; 31.1% healthcare workers; 41.5% general servicemen. (95.8%)
Quinn et al.^27^	Cross‐sectional study	Pandemic influenza A(H1N1)	USA (June–July 2009)	N=1543 (46.3)	Members of US general public aged 18 or over (48.2%)
Rubinstein et al.^19^	Semistructured interviews and focus groups with samples of the general public	Hypothetical pandemic scenarios	London and Southampton (November 2013–March 2014)	N=71 (16–35, n=21 (29.6%); 36–64, n=20 (28.2%); 65+ y, n=30 (42.3%)	Diverse samples of general public with different at‐risk status (32.4%)
Seale et al.^35^	Cross‐sectional study	Influenza pandemic	Sydney, Australia (June–October 2007)	N=1079 (18–30, n=338 (31.3%); 31–40, n=280 (25.9%); 41–50, n=247 (22.9%); 51+, n=186 (17.2); not specified, 28 (2.6%)	Healthcare workers (22.7% male; 2.5% unspecified)

**Table 4:** Results of studies measuring actual adherence

Citation	Outcome measures (self‐report unless stated otherwise)	Adherence with oseltamivir (calculated as a % of those prescribed antivirals unless otherwise noted)	Predictors of adherence (significant results in bold)	Self‐report reasons for non‐adherence	Other	Risk of bias
Belmaker et al.^15^	Adherence. Reasons for discontinuation	87.6% (176/201). Post‐exposure group = 85% adherence	Age, sex, exposure type (post‐/pre‐exposure). Additional in post‐exposure group: interval between estimated exposure and beginning oseltamivir, side effects of oseltamivir, reasons for non‐adherence.	Did not receive enough tablets (n=5), side effects (n=4), chose not to take oseltamivir (n=4).	—	C
				In the post‐exposure group (6/40): side effects of dizziness and diarrhoea (n=1); did not start because no direct contact with infected poultry (n=1); not reported (n=4)		
Chishti & Oakeshott^32^	Adherence. Reasons for discontinuation	82% (27/33) Took oseltamivir for less than 5 d (n=4); did not start the medication at all (n=2)	—	Reasons for not beginning course of oseltamivir: clinical improvement (n=1), fear of side effects (n=1).	—	B, C, D
				24% (8/33) reported side effects attributed to oseltamivir		
Choo et al.^38^	Adherence. Reasons for discontinuation	“11.2% (37/331) non‐adherent” (paper does not give figure for total adherent)	Age, sex, ethnicity dialysis treatment type, dialysis centre, side effects (presence and persistence), forgetting to take medication, fear, not wanting to take oseltamivir	56.8% (n=21) forgot to take pill; 24.3% (n=9) perceived side effects; 8.1% (n=3) did not want to take oseltamivir; 5.4% (n=2) lost pills; 2.7% (n=1) were fearful; 8.1% (n=4) other reasons	—	C
Coleman et al.^18^	Adherence, objective recording by pill counts at each visit and electronic medicine vial caps	30% (13/43) NB—adherence defined as taking 6–7 pills per week (pills should be taken daily)	Personal, household and work‐related variables, symptoms of and contact with respiratory illness, **earlier time in study (86 vs 75 pills per 100 person‐days of follow‐up; ** ***P***=**.001)**	4/42 (10%) discontinued oseltamivir before 13 wk due to adverse effects	Participants took oseltamivir for 5–155 d (median 121).	A, C, D
Griffiths et al.^39^	Adherence. Opinions on preventive measures	70.2% “47.6% of all students picked up Tamiflu (all were prescribed it without cost)”	**Gender (OR** = **2.43, 95% CI [1.26–4.71], ** ***P*** **<.001)**, age, **country of study (OR** = **2.68, 95% CI [1.04–6.91], ** ***P*** **<.05),** HK residency, **compliance with guidelines prior to arrival (offline, practice‐based guidelines. OR** = **1.19, 95% CI [1.07–1.33], ** ***P*** **<.001)**,** opinions on necessity of measures (temperature checkpoints. OR** = **0.79, 95% CI [0.67–0.94], ** ***P*** **<.001; contingency measures, OR** = **1.16, 95% CI [1.05–1.29], ** ***P*** **<.001)**	—	—	—
Kimberlin et al.^33^	Adherence. Incidence of adverse events	44% (29/65) Mean number of missed doses: counsellors = 1, staff = 1 (range 0–4)	Adverse events (presence, number), employment status (staff/counsellor)	—	—	B, D
Kitching et al. (2009)^23^	Adherence. Incidence of adverse effects	66% (56/85). 89% (n=85) schoolchildren took any oseltamivir	—	Parents reported negative comments on oseltamivir efficacy.	56/85 (55%) of those who took “any oseltamivir” completed/would complete a full 10‐d prophylaxis course.	B, C, D
				Parents sceptical of need for mediation if indication was to prevent onward transmission (vs giving specific benefit to individual asymptomatic child)		
				Many parents questioned basis of scientific basis or advice.		
				Risk of oseltamivir (potential side effects) perceived as higher than “risk” of influenza.		
Koliou et al.^24^	Adherence	88.2% (15/17)	—	Incidence of adverse events		A, B, C, D
Lonsdale & Way^25^	Adherence. Incidence of adverse effects	86% took oseltamivir for 5 d or more.	—	Reasons for non‐adherence after having started course: symptoms disappeared/they were better, suffered side effects, afraid of side effects, thought did not have influenza	Worried about side effects, not having influenza (confirmed or suspected), feeling better).	A, B, C
		Not starting course of medication (n=8).				
McVernon et al.^34^	Adherence to behavioural and pharmaceutical recommendations.	75% (95% CI [68.2, 80.6%]).7.1% refused it altogether; 9.9% took up to half; 5.1% more than half; 2.9% were unsure.	Age, the presence of a case in the household, and attitudes to the intervention, compliance of all family members with behavioural recommendations and pharmaceutical interventions in quarantine period	Where non‐compliant, primary household respondent attributed this to belief that drug was unnecessary (n=42), particularly for individuals older than 18 (*P*=.02).	—	B
Rajatonirina et al.^28^	Adherence. Incidence of adverse effects	Overall, 61.4%. Treatment: 90% (18/20; 4/20 took a prolonged course according to associated risk factors e.g. asthma). Prophylaxis: 56.2% (63/112). 8/112 (7.1%) did not take oseltamivir	Sex, report of illness in weeks before/during school closure, individual symptom associated with adherence (headache, fatigue, difficulty concentrating, sleeping sickness), adverse events	Forgetting (n=22), not feeling sick (n=15). Eight did not take any doses: did not feel sick, n=3; worried about taking it, n=2; did not like taking it, n=1; did not specify any reason, n=2.	Oseltamivir as prophylaxis: 75/112 (66.9%) took medication for at least 1 wk.	A, B
Simonsen et al.^37^	Use of health preventive measures	39% (140/357) reported oseltamivir use	**Age (≤40 y old [44% vs 31%], ** ***P***=**.021).**	—	—	B, C
Skowronski et al.^16^	Adherence. Duration of course	59% (44/75) (Direct exposure group, 63%; 40/64)	Age, sex, province of residence, occupation, epidemiological data (**direct contact with infected poultry (64/90 [71%] vs 10/75 [13%]; OR** = **16, 95% CI 6.7–39.2)**)	—	Took oseltamivir for treatment, 5% (n=4); for prevention, 95% (n=70)	A, B
Strong et al.^29^	Adherence. Incidence of adverse effects. Incidence of flu symptoms	Did not complete full course of oseltamivir = 15% (48/326). Accepted offer of taking oseltamivir. Students, 92% (273/297); staff, 91% (53/58).	Whether or not respondent had been “poorly with flu symptoms,” which symptoms experienced, any past medical history, **adverse effects (nausea**,**OR** = **2.4; 95%CI 1.2–4.9%, ** ***P***=**.013; vomiting, OR** = **3.5, 95%CI 1.3–9.3, ** ***P***=**.014; rash, OR** = **13.0, 95%CI 1.1–299.3, ** ***P***=**.046),** pupil/staff	Tablets made them feel unwell, 50%; didn't think they would help, 17%; forgot to take tablet, 15%	—	—
Upjohn et al.^30^	Adherence	72.2% (26/36)	Age, sex, HCW role, exposure, adverse events. Knowledge and beliefs about transmission and prevention of influenza (**discussed with someone taking oseltamivir without problems, (10/10 [100%] vs 4/9 [44.5%], ** ***P***=**.02).**	Adverse events, n=5 (50%), index patient found not to have influenza, n=3 (30%);	One did not start course because of concerns about adverse effects. 27.8% (n=10) did not complete course ‐ stopped at median of 3 d.	A, B, C, D
Ushijima et al.^17^	Adherence	79% (179/228)	Age, sex, reasons for discontinuation	Relief of symptoms (n=31); adverse events, n=9; refusal to take drug, n=5	—	A, B, C
Van Velzen et al.^36^	Adherence	Overall adherence ‐ 79% (95% CI [70,87%])	**Lower daily dose (prophylaxis lower dose 85% vs treatment higher dose 61%, ** ***P***=**.01)**	Child had difficulty swallowing medication, n=6; medical advice to stop the course, n=5; parents did not see (further) benefit, n=3; occurrence of adverse effect, n=2	—	D
Wallensten et al. 31	Adherence	77.2% (190/247). One child (0.4%) did not take any doses.	Sex, incidence of side events, illness in the week before/during school closure	Tablets made them feel unwell, n=24; forgot to take tables, n=22;	91.9% (227/247) took oseltamivir for at least 7 d.	A, C

A—selection bias; B—detection bias; C—reporting bias; D—other sources of bias.

**Table 5:** Results of studies measuring intended adherence

Citation	Outcome measures	Intended adherence with oseltamivir	Predictors of adherence (significant results in bold)	Risk of bias
Bults et al.^21^	Perceived risk, feelings of anxiety, behavioural responses	Probably/certainly intend to take antiviral medication. Survey 1, 82%; Survey 2, 76%; Survey 3, 3.1, 71%; 3.2, 70%	Univariate analysis: Knowledge, risk perception (**perceived severity, perceived vulnerability**, perceived anxiety), response efficacy (**efficacy of measures, underestimation and fatalism statements**), self‐efficacy, maladaptive responses, **amount of information received, reliability of governmental information.**	—
			Multivariate analyses: **No children in the household (OR** = **1.45, 95% CI [1.04–2.0], ** ***P***=**.03), high anxiety (OR** = **1.93, 95% CI [1.43–2.61], ** ***P*** **<.001), higher level of self‐efficacy (OR** = **1.68, 95%CI [1.26–2.22], ** ***P*** **<.001), more in agreement with statements on avoidance (OR** = **1.43, 95% CI [1.07–1.90], ** ***P***=**.02), paying much attention to information on pandemic influenza (OR** = **2.36, 95%CI [1.67–3.33], ** ***P*** **<.001)**	
Ibuka et al.^22^	Completing a course of antiviral medication	57.1% would take antivirals as prevention for pandemic. 83.2% would take antivirals as treatment for pandemic	Age, **female sex (cure, β** = **0.39, ** ***P***=**.02), larger household size (cure, β** = **0.14, ** ***P***=**.03**), risk perception (**perceived likelihood of getting swine flu, prevention, β** = **1.83, ** ***P*** **<.001; cure, β** = **1.82, ** ***P*** **<.001**), estimated death toll, **engagement in information seeking activities, prevention, β** = **0.78, ** ***P*** **<.001; cure, β** = **1.03, ** ***P*** **<.001),** willingness to accept pharmaceutical intervention, precautionary behaviours (**taking quarantine measures, prevention, β** = **0.84, ** ***P***=**.02**), geographical risk status (number of H1N1 cases in respondent's state), time in survey.	B
Lynch et al.^20^	Likelihood to take oseltamivir while pregnant	43.5% (61/140) likely or somewhat likely to take antivirals, like oseltamivir, for flu while pregnant	**Unfamiliarity with antiviral medicine, pandemic flu (vs seasonal), concerns about potential side effects for foetus**	A
Masuet‐Aumatell et al.^26^	Intention to comply with influenza antiviral as either prophylaxis or treatment	58.3% intend to take antiviral drug as preventive measure for influenza. 74.2% intend to take antivirals as therapeutic measure for influenza	Sex, **age (therapy, ≥40 y, 80.6% vs 9.4%, ** ***P***=**.015)**, physical status, drug consumption, underlying condition, **dispensed antimalarial prophylaxis (therapy, 81.3% vs 58.1%, ** ***P***=**.008)**, vaccination prescribed, prior travelling, travel purpose, duration of stay, destination. **Lack of knowledge of influenza prevention (prevention, 84.0% vs 66.6%, aOR** = **0.14, 95% CI [0.03–0.65]), knowledge of potential risks of infection (therapy, 78.6% vs 33.3%, aOR** = **7.02, 95% CI [1.11–44.21]). Positive attitude towards influenza prevention (prevention, 74.5% vs 36.6%, aOR** = **4.26, 95%CI [1.54–11.73). Motivation e.g. considering carrying antivirals while travelling (prevention, 70.2% vs 31.0%, aOR** = **4.66, 95% CI [1.52–14.30])**	—
Yap et al.^14^; Phern‐Chern, personal communication	Oseltamivir as effective treatment and prevention for pandemic influenza	85.5% (970/1134) would complete a course of oseltamivir if prescribed for pandemic influenza	Univariate analysis: Female sex, older age group, exposure group (patients, contacts, HCW vs general individuals), **ethnicity (Malay, Indian vs Chinese),** education, role in military, private housing, adverse events, **higher knowledge score,** practice score	B, C
			Multivariate analysis: sex, age, exposure group patients, **Malay ethnicity (β** = **3.30, 95% CI [0.67–5.92, ** ***P***=**.014), higher knowledge scores (β** = **0.21, 95% CI [0.14–0.28], ** ***P*** **<.001),** practice score	
Quinn et al.^27^	Attitudes towards pandemic influenza. Willingness to accept an EUA drug	54.5% (869/1519) probably/definitely would accept Tamiflu for self. 48.8% (276/521) probably/definitely would accept Tamiflu for child.	Refusal to accept vaccine. **Ethnicity – accept drug for self (** ***P*** **<.01) and child (** ***P*** **<.05). Age (18–34) – accept drug for child (** ***P*** **<.001). Prior history of having flu vaccine ‐ accept drug for self (** ***P*** **<.001).** Knowledge and attitudes towards pandemic. Language from a CDC factsheet. **Perceived personal consequences ‐ accept drug for self (** ***P*** **<.05). Trust in government ‐ accept drug for self (** ***P*** **<.001) and child (** ***P*** **<.05).** Healthcare status. **Worry about EUA drug – accept drug for self (** ***P*** **<.001) and child (** ***P*** **<.001). Dispensed by non‐health professional. Lower level of education (when dispensed by public health professional; ** ***P***=**.01)**	B
Rubinstein et al.^19^	Knowledge and experiences of antiviral medication. Responses to uncertain scenarios concerning antivirals	Not reported	**Capability (psychological). Motivation (automatic and reflective). Opportunity (physical and social).**	—
Seale et al.^35^	Likelihood of taking course of antivirals (oseltamivir/zanamivir)	81.3% (n=877) intend to take course as instructed. 6.8% (n=73) would divert medications to family members	Take antivirals for self. Medical/nursing staff. **Comply with quarantine measures (OR** = **1.93, ** ***P*** **<.05).**	B, C
			Divert antivirals to family (all/some). **Female sex (OR** = **0.49, ** ***P*** **<.05), have children (OR** = **1.81, ** ***P*** **<.05), medical/nursing (OR** = **0.46, ** ***P*** **<.05), ancillary/support (OR** = **1.06, ** ***P*** **<.05), very unhappy about quarantine (OR** = **1.76, ** ***P*** **<.05)**	

A—selection bias; B—detection bias; C—reporting bias; D—other sources of bias.

### 3.2 Assessment of risk of bias

We identified serious methodological flaws in several of the included studies. Studies showing higher risk of bias (selection bias, detection bias, reporting bias and other sources of bias) are identified in Table 4 and 5. Many studies did not clearly report response rates^16^, ^17^, ^18^, ^20^, ^21^, ^22^, ^23^, ^24^, ^25^, ^26^, ^27^, ^28^, ^29^, ^30^, ^31^ or eligibility criteria to ensure that the sample was representative.^14^, ^16^, ^17^, ^19^, ^20^, ^24^, ^25^, ^28^, ^30^, ^31^, ^32^, ^33^, ^34^, ^35^, ^36^ Additionally, four studies had small sample sizes.^18^, ^24^, ^30^, ^32^ Multiple studies lacked clearly defined outcomes^14^, ^16^, ^17^, ^18^, ^22^, ^23^, ^24^, ^25^, ^27^, ^28^, ^30^, ^32^, ^33^, ^34^, ^35^, ^37^ and all but two studies^18^, ^19^ lacked valid and reliable methods of outcome measures, both factors contributing to higher potential detection bias. Reporting bias was found in studies that did not report confidence intervals^15^, ^17^, ^18^, ^23^, ^24^, ^25^, ^30^, ^31^, ^32^, ^35^ and which did not carry out appropriate analyses of association between adherence rates and potential predicting factors.^17^, ^23^, ^24^, ^25^, ^31^, ^32^, ^37^, ^38^ Other sources of bias included the fact that none of the studies reported a sample size calculation. Main potential confounders were not taken into account in ten studies.^17^, ^18^, ^23^, ^24^, ^25^, ^29^, ^31^, ^32^, ^33^, ^37^ Fourteen studies also did not address appropriate or clearly focused questions.^14^, ^16^, ^17^, ^18^, ^23^, ^24^, ^25^, ^29^, ^30^, ^31^, ^32^, ^33^, ^36^, ^37^

### 3.3 Actual adherence

In studies investigating actual adherence with oseltamivir, adherence ranged from 30% to 88.8%.^38^ Most studies reported a high overall adherence in the range of 70%–89%. Eight studies investigated adherence to antivirals only as a preventative measure (six in the context of influenza A(H1N1)pdm09), three investigated adherence to antivirals only as a therapeutic measure (two in relation to influenza A(H1N1)pdm09), five investigated predictors of adherence to antivirals as both treatment and prophylaxis (all in reference to influenza A(H1N1)pdm09), and two studies did not report the reason for antiviral prescription.

#### 3.3.1 Antivirals as prophylaxis

When antivirals were prescribed as prophylaxis for influenza A(H1N1)pdm09, the only demographic predictor of adherence was sex, with male students being more likely to take oseltamivir than female students.^39^ Other predictors included an earlier time in the pandemic,^18^ country of study (higher adherence in students studying in Singapore than those studying in the United States),^39^ previous compliance with other precautionary advice about pandemic flu,^39^ beliefs that the recommended preventative measures were necessary^39^ and having discussed taking oseltamivir with someone who had not experienced side effects.^30^ Where antivirals were prescribed as prophylaxis for avian influenza (H7N3), having had direct contact with infected poultry was a significant predictor for adherence to antivirals.^16^

Where self‐reported reasons for non‐adherence to antivirals as prophylaxis for influenza A(H1N1)pdm09 were stated, occurrence of adverse events was the most commonly reported reason for the discontinuation of oseltamivir.^18^, ^30^, ^31^, ^38^ Fear (cause unspecified) was also given as a reason for discontinuation of oseltamivir in end‐stage renal failure patients,^38^ as was not wanting to take it.^38^ For those taking oseltamivir in conjunction with their work, there being no direct contact with the virus was given as a reason for non‐adherence.^30^ Forgetting to take oseltamivir^31^, ^38^ and losing tablets^38^ were also given as reasons for discontinuation.

Where self‐reported reasons for non‐adherence to antivirals as prophylaxis in non‐pandemic situations were presented, the presence of adverse events was given as a reason for discontinuation of antivirals for influenza H5N1^15^ as was not receiving enough tablets.^15^ For those taking oseltamivir as a result of avian influenza outbreak (H7N3) at work, not being in direct contact with the virus was a reason given for discontinuation of oseltamivir.^16^

#### 3.3.2 Antivirals as treatment

No predictors of adherence were found when antivirals were prescribed only as treatment for influenza A(H1N1)pdm09, nor were any statistically significant predictors of adherence found in non‐pandemic situations.

Where self‐reported reasons for non‐adherence to antivirals as treatment for influenza A(H1N1)pdm09 were stated, occurrence of adverse events was the most commonly reported reason for the discontinuation of oseltamivir.^24^, ^25^ The fear of developing side effects was also sufficient to stop people from beginning oseltamivir. Perception of clinical improvement was given as a self‐reported reason for stopping treatment with oseltamivir. Self‐reported reasons for non‐adherence to antivirals as treatment in non‐pandemic situations (seasonal influenza) included the presence of adverse events.^15^

#### 3.3.3 Antivirals as treatment and prophylaxis

One study identified taking a lower, prophylactic, daily dose rather than a higher, treatment daily dose as being a predictor of adherence to antivirals for influenza A(H1N1)pdm09.^36^ Two studies investigating antivirals prescribed both as treatment and as prophylaxis for influenza A(H1N1)pdm09 identified significant predictors of adherence, but unfortunately the studies did not differentiate between predictors of adherence to antivirals as treatment vs prophylaxis. In these cases, the presence of adverse effects,^29^ age, with higher adherence to antivirals for influenza A(H1N1)pdm09 in those aged <40, and the presence of adverse effects were identified as predictors of adherence.

Self‐reported reasons for discontinuation of antivirals for influenza A(H1N1)pdm09 in studies that did not differentiate between treatment and prophylactic use of antivirals included the presence of adverse events^29^, ^36^ as well as the perception that antivirals were not effective.^23^, ^29^ People also stopped taking oseltamivir because they had medical advice to stop taking the drug,^36^ forgot to take it^28^, ^29^ and because they had difficulty swallowing the tablets.^36^ The perception that there was a greater risk of developing side effects after having taken oseltamivir than there was a risk of catching influenza A(H1N1)pdm09^23^ also stopped people from beginning to take antivirals for influenza A(H1N1)pdm09.

Studies which did not specify at all the reason why antivirals were prescribed as treatment or prophylaxis for influenza A(H1N1)pdm09 also identified fear of developing side effects as a reason not to begin oseltamivir and perception of clinical improvement as a reason for stopping oseltamivir.

### 3.4 Intended adherence

Studies investigating predicted adherence with antivirals found an intended adherence rate ranging from 43.5%^20^ to 85.5% (Phern‐Chern, personal communication). All studies reported intended adherence to antivirals in pandemic situations, but one also investigated hypothetical seasonal flu situations.^20^ All studies found significant predictors of adherence. Female sex was associated with higher intended adherence to antivirals for oneself^22^ and intention to divert antivirals to family members.^35^ Older age was also predictive of adherence^26^, ^27^ as was Malay ethnicity in the Singaporean military^14^ and ethnicity in the acceptance of antivirals for the self and for one's children in the US population.^27^ Household composition was also associated with intended adherence to antivirals in the general population, but with opposing results in different countries. In the United States, larger household size was a predictor of intended adherence to antiviral medication for the treatment of pandemic influenza,^22^ whereas in the Netherlands, intention to take antivirals as prophylaxis was associated with not having children in the household.^21^ Other predictors of intended adherence to antivirals included higher risk perception of catching pandemic influenza,^21^, ^22^ knowledge of pandemic influenza^14^ and its associated risks^26^ and perceived personal consequences of influenza^27^ as well as amount of information received about pandemic influenza,^21^ increased attention to information^21^ and information seeking behaviours.^22^ In the United States, trust in the government was also associated with acceptance of antivirals for oneself and one's children.^27^ Higher levels of anxiety, self‐efficacy and response efficacy,^21^ worry about antivirals^27^ and a positive attitude towards influenza prevention^26^ were also predictive of intended adherence to antivirals, as were compliance with other preventive measures^22^, ^26^, ^35^ and having previously accepted the flu vaccine.^27^

Two studies investigating intended adherence used qualitative methods. The first investigated pregnant and recently pregnant women's perceptions about both seasonal and pandemic influenza.^20^ They found that unfamiliarity with antiviral medicines, concerns about potential side effects for the foetus and the pandemic nature of influenza (over seasonal influenza) were predictors for adherence to antiviral medication. The second study^19^ reported the views of diverse samples of the general public with different at‐risk status within the framework of the COM‐B model of behaviour change.^8^ Within this context, psychological capability (e.g. knowledge of the pandemic), automatic motivation (e.g. being vaccinated for seasonal flu), reflective motivation (e.g. beliefs about the effectiveness and safety of the medicine, health identity), physical opportunity (e.g. access to treatments and professional advice) and social opportunity (e.g. trust in recommendations from health professionals) were identified as being important for adherence to a course of antiviral medication.

## 4 Discussion

Identifying predictors of adherence can help to inform areas which can be targeted to improve adherence further still. Attempts to identify demographic predictors of adherence have given mixed results, with male sex being found as a predictor for actual adherence in summer school students^39^ and female sex being found as a predictor of intended adherence in the Singaporean military.^22^ Age was also a predictor of actual adherence with oseltamivir during the 2009/2010 influenza pandemic, with higher adherence seen in people with an influenza diagnosis in Norway aged under 40.^37^ In contrast, older age was found to predict intended adherence to antivirals amongst a representative sample of the US population^27^ and travellers attending a UK travel clinic.^26^ This may be because older adults are at a higher risk of developing complications from influenza and have a higher probability of having previously had the flu vaccine, and so this portion of the population may be more used to accepting pharmaceutical interventions as personal protection.

There were no major qualitative differences between predictors of adherence to antivirals as treatment or prophylaxis for influenza. Any minor differences are likely because prophylactic courses of antivirals tended to be longer than treatment courses, with one study in particular indicating a 20‐week prophylactic prescription period.^18^ Due to the lack of major qualitative differences between predictors, results for predictors of adherence to antivirals as treatment and prophylaxis are discussed together in the context of the wider literature. Furthermore, only three studies investigated predictors of actual adherence to antivirals and self‐reported reasons for discontinuation of antivirals in non‐pandemic influenza situations.^15^, ^16^, ^17^ The results of these three studies did not differ qualitatively for pandemic and non‐pandemic influenza and results from pandemic and non‐pandemic studies are therefore also discussed together.

The most commonly found predictor of actual and intended adherence, and reason given for non‐adherence, was the incidence of adverse side effects, with studies indicating that the fear of side effects was enough to stop people from beginning a course of oseltamivir. Members of the public may perceive the incidence of side effects from antivirals to be considerably higher than is actually the case. According to clinical trial data, the most common side effect of oseltamivir as a treatment for influenza is nausea (without vomiting) affecting 10% people, with the most common side effect of oseltamivir when used as prophylaxis being headache, affecting 18% people. However, approximately half the Singaporean military surveyed perceived adverse side effects to be caused by oseltamivir (Phern‐Chern, personal communication). Correcting misperceptions about the rates of side effects associated with oseltamivir may help to boost rates of adherence.

Other non‐demographic predictors of adherence largely fell into categories identified by the COM‐B model of behaviour change^8^(see Table 6), in line with results from Jackson et al.^40^ The COM‐B model of behaviour change identifies three components—capability, motivation and opportunity—all of which are necessary to target when attempting to initiate behaviour change. Capability can be further divided into physical and psychological capability, motivation into reflective and automatic motivation, and opportunity into physical and social opportunity.^8^ With reference to the particular predictors of adherence identified in this review, knowledge about influenza, amount of information about influenza and increased attention to information about influenza could be categorised within psychological capability. For example, one predictor found to be associated with adherence to antivirals was knowledge about pandemic flu. Although knowledge about influenza A(H1N1)pdm09 was moderate,^41^ there have been calls for education about oseltamivir because it being confused with a vaccine for influenza.^42^ Analysis of the content of one year's worth of UK newspaper articles relating to the 2009/2010 influenza pandemic found that approximately 10% of articles mentioned that oseltamivir helped symptoms of influenza A(H1N1)pdm09.^43^ This proportion is relatively low, potentially affecting peoples' perceptions of oseltamivir, in turn affecting compliance rates. For this reason, it may be important to educate the general public about the risks of influenza, both seasonal and pandemic as well as ensuring clarity when delivering messages about who is recommended for pharmaceutical interventions.

**Table 6:** Predictors of uptake of antivirals identified in relation to the COM‐B model of behaviour change

Capability	Motivation	Opportunity
*Psychological* Knowledge of the virus Amount of information received about the virus Information seeking behaviours Increased attention to information about virus Forgetting	*Reflective* Perception of virus and associated risks Perception of antivirals and associated risks (response efficacy) Belief of necessity of precautionary behaviour Self‐efficacy Perceived personal consequences of flu Positive attitude towards prevention of flu	*Physical* Losing pills Not having enough pills
*Physical* Difficulty swallowing pills	*Automatic* Habitual behaviour of previous compliance with precautions Emotion—fear (of antivirals, and of side effects of antivirals) Emotion—anxiety	*Social* Trust in government Speaking to someone who has experienced side effects previously

Risk perception, including perceived severity of and vulnerability to the outbreak, was also found as a predictor of intended adherence to antiviral medication. When communicating with the public, the perception of the overall severity of the outbreak should not be overlooked, as it influences the adoption of behavioural changes.^44^ It has been suggested that during the pandemic, the general public were complacent and passive, making them vulnerable to influenza A(H1N1)pdm09 through their lack of uptake of safety behaviours.^45^

In terms of motivation, perception of the risk of catching influenza, perception of the severity of influenza, perceived personal consequences of influenza, attitude towards the prevention of influenza beliefs associated with the necessity of precautionary behaviours and concern about the safety, side effects and effectiveness of the pharmaceutical recommendation could be classified as reflective motivation. Automatic motivation comprises emotions of fear (towards antivirals themselves and side effects elicited by antivirals), and higher anxiety levels, as well as previous engagement in precautionary behaviours including previous acceptance of the influenza vaccine. Social opportunity includes predictors such as trust in the government and speaking to someone who had previously experienced side effects when taking antivirals and physical opportunity incorporates losing the pills or not having enough pills. The psychological capability aspect of the COM‐B model is reflected in forgetting to take the pills, whereas the physical capability aspect could be seen in having difficulty swallowing them.

Predictors that are less easy to categorise within the COM‐B model were also identified. One study identified time in the study period as a predictor of adherence to oseltamivir with participants being more adherent in the first 10 weeks of the study than in the second 10 weeks. This is possibly due to changing perceptions of the risk of contracting flu as the pandemic season continued and concern about the effects of long‐term prophylaxis (both linked to reflective motivation in the COM‐B model) as well as the increasing burden in everyday life of continuing to take oseltamivir prophylaxis daily (linked to physical capability in the COM‐B model). Higher adherence with lower daily doses of oseltamivir was also found as a predictor of adherence and could be due to the perception that taking less of a drug is safer and better tolerated, but this speculative interpretation should be treated with caution.

### 4.1 Limitations of the reviewed literature

Overall, the studies reviewed left room for improvement in methodological rigour. Of the 26 included studies, nine showed high risk of selection bias, fifteen showed high risk of detection bias, fourteen showed high risk of reporting bias and seven showed high risk of other sources of bias (see Tables 4 and 5). Studies of particular concern had small sample sizes (<60 participants),^18^, ^24^, ^30^, ^32^ limited robustness of outcome assessment and no analysis of association between adherence rates and potential predicting factors.^17^, ^23^, ^24^, ^32^ These sources of bias could have had a profound impact on the results of the individual studies. For instance, the fact that no studies reported having conducted a power calculation suggests that predictors of adherence that may exert medium to small effects may have been missed.

Other methodological limitations of the studies reviewed include that that self‐report measures of adherence were used in all but one of the 26 studies reviewed, in which objective measures of adherence such as pill counts and the use of electronic medicine vial caps were used. In general, self‐report measures of adherence tend to result in higher estimates of adherence compared with objective measures.^46^, ^47^

Although we did not systematically search for rates of adherence to antivirals and therefore any interpretation of adherence rates should be taken with caution, it is interesting to note that in some cases intended adherence rates to antivirals were lower than actual adherence. This is surprising as although people may intend to carry out a particular health behaviour, they do not necessarily always do so; this is termed the “intention–behaviour gap”.^48^ However, an explanation for this pattern may lie in the populations of participants assessed in the studies reviewed. Many of the studies reporting actual adherence to oseltamivir were conducted in specified populations who were prescribed oseltamivir as part of their job, either to protect themselves from catching influenza (e.g. poultry farmers^15^, ^16^) or to protect already at‐risk patient populations (e.g. healthcare workers^18^, ^30^). Other studies included populations of people who were already ill or at‐risk from influenza (e.g. renal patients^38^) or who had been in close contact to a confirmed case of pandemic influenza (school studies^23^, ^29^, ^31^, ^33^, ^36^, ^39^), with all participants enrolled in these studies having already been prescribed antivirals.

Conversely, three studies investigating intended adherence to oseltamivir were conducted in samples of the general population in different countries (representative samples of the Netherlands^21^ and United States^22^, ^27^). Amongst these participants, there will be a large proportion of people for whom antivirals would not be prescribed in the hypothetical scenarios set out by the studies, or who may fit into “at‐risk” populations but who may be unaware of the recommendations relating to antivirals.^49^, ^50^ There may also be a number of people who display symptoms of flu, but who do not present to health care providers. Such people would not be identified or included in research investigating actual adherence rates to antivirals. In this way, studies investigating actual adherence in the subset of patients who present to primary care may overestimate likely adherence in the general population.

The difference between adherence defined as collecting and then finishing a course of antivirals, and adherence defined as completing a course once the first tablet has been taken, can also be demonstrated by one of the papers in this review.^39^ In this study, all students attending a summer school were offered oseltamivir without cost. Of these students, only 47.6% students picked up oseltamivir, of whom 70.2% took the medication. This means that 33.4% of students took oseltamivir, a much lower number than an adherence rate calculated from only those who picked up the drug.

### 4.2 Limitations of the review

To the best of our knowledge, this is the first systematic review investigating predictors of antiviral adherence. One particular strength of this review is that studies were not limited only to influenza A(H1N1)pdm09, but also included predictors of adherence of antivirals during seasonal influenza, and so can help inform routine communications with the public.

The limitations of this systematic review are inherently bound to the methodologies of the original research studies reviewed. Studies used very different methodologies to assess adherence and predictors of adherence to antivirals in a wide range of participant populations. In addition to this, differences in definitions of adherence and length of recommended use of antivirals (spanning 5 days to 20 weeks) between studies further limited interpretation of results. Although the presence of these factors has broadened the results, with predictors related to time within the study being identified, the influence of these factors made comparisons between studies difficult. Due to this wide variation in studies reviewed, meta‐analysis of results was not appropriate. The poor reporting of effect sizes for individual studies was also problematic.

Another limitation of the review is that the neither the grey literature, nor unpublished papers were searched for pragmatic reasons, nor did we undertake forward citation tracking; articles not written in English and conference abstracts were also excluded. This increases the likelihood that some research into predictors of adherence to antivirals has been overlooked. The nature of publication bias, in which studies finding a statistically significant result are more likely to be published, suggests that any literature that we missed is unlikely to have found significant predictors of adherence.

One further thing to consider when interpreting the results of this review is that there was a wide variety of populations included, from poultry workers, to renal patients and that results were not subcategorised according to population type. Had these further distinctions been made, there would have been few articles in each group.

## 5 Conclusions

The most consistently found predictor of actual or intended adherence and reason given for non‐adherence to antivirals was the incidence of side effects. Other predictors can help to inform future strategies to increase adherence to antivirals. In particular, increasing knowledge about the risks of influenza, correcting misperceptions about side effects and putting into perspective the risks of antivirals may help to increase future adherence to antivirals.
