# Supplementary material for: A systematic review of factors affecting intended and actual adherence with antiviral medication as treatment or prophylaxis in seasonal and pandemic flu
Source: Influenza Other Respir Viruses. 2016 Aug 8;10(6):462–78. doi: 10.1111/irv.12406 (PMC5059947; doi:10.1111/irv.12406)
Supplement: Supplementary file 1 [file IRV-10-462-s001.docx]

Supplementary Item 1. Search strategy conducted in Embase; similar searches conducted in other databases.

| 1 | (antiviral agents or anti-viral?) |
| --- | --- |
| 2 | antiviral |
| 3 | tamiflu |
| 4 | relenza |
| 5 | oseltamivir |
| 6 | zanamivir |
| 7 | (neuraminidase and inhibit*) |
| 8 | 1 or 2 or 3 or 4 or 5 or 6 or 7 |
| 9 | (adherence or compliance or uptake) |
| 10 | pandemic |
| 11 | (influenza or flu or h1n1 or h5n1) |
| 12 | 10 or 11 |
| 13 | 8 and 9 and 12 |
